# The Role of the Nervous System in Lung Disease

**DOI:** 10.1007/s11910-026-01511-4

**Published:** 2026-09-16

**Authors:** Connar M. A. Mawer, Archie Patel, Nadja Zeltner, Marilyn K. Glassberg Csete, Erica L. Herzog

**Affiliations:** 1 Pulmonary, Critical Care, and Sleep Medicine, Department of Internal Medicine, Yale School of Medicine, New Haven, CT 06520 USA; 2 Center for Molecular Medicine, University of Georgia, Athens, GA 30602 USA; 3 Department of Biochemistry and Molecular Biology, University of Georgia, Athens, GA 30602 USA; 4 Department of Cellular Biology, University of Georgia, Athens, GA 30602 USA; 5 Stritch School of Medicine, Department of Medicine, Loyola University, Chicago, Maywood, IL 60153 USA; 6 Department of Pathology, School of Medicine, Yale University, New Haven, CT 06520 USA

**Keywords:** Lung, Nerves, Pulmonary fibrosis, Asthma, Lung cancer, hiPSCs, Pluripotent stem cells

## Abstract

**Purpose of Review:**

Peripheral nerves regulate pulmonary physiology in a nerve-type- and compartment-specific manner through neurotransmitters, neurotrophins and axon-guidance cues. This review examines how sensory and autonomic pathways influence pulmonary homeostasis and disease, and evaluates the experimental models available to investigate these interactions.

**Recent Findings:**

Dysregulated pulmonary innervation has been implicated in acute lung injury, airway hyperresponsiveness, pulmonary fibrosis and lung cancer, where neural signals may modulate immune activation, stromal remodelling and tumour progression. However, pulmonary neurobiology remains comparatively underexplored, in part because neuronal cell bodies are located outside the lung and pulmonary axons and nerve terminals are sparse, fragile and difficult to resolve using conventional methods. Rodent models remain essential for establishing causal mechanisms within intact neural circuits, although species-specific differences limit direct translation. Human pluripotent stem cell-derived sensory and autonomic neurons offer a platform for dissecting human nerve-immune and nerve-stromal interactions.

**Summary:**

Integration of mechanistic animal studies with human stem cell-derived neuronal models may overcome current experimental limitations and provide a more physiologically relevant framework for defining the contribution of pulmonary nerves to respiratory disease.

## Introduction

Pulmonary function is governed by a dynamic neuronal network, involving both central and peripheral systems. The central nervous system (CNS) circuits within the brainstem, including the ventral respiratory column, generate and regulate the respiratory breathing rhythm [1], and within the lung, peripheral nervous system (PNS) pathways modulate airway tone, immune responses, and local tissue homeostasis. Given the broad functional roles of the PNS in lung physiology, dysregulation of these nerves is increasingly recognised as a key contributor to lung disease [2–6].

## Pulmonary Neurophysiology

**Fig. 1:**
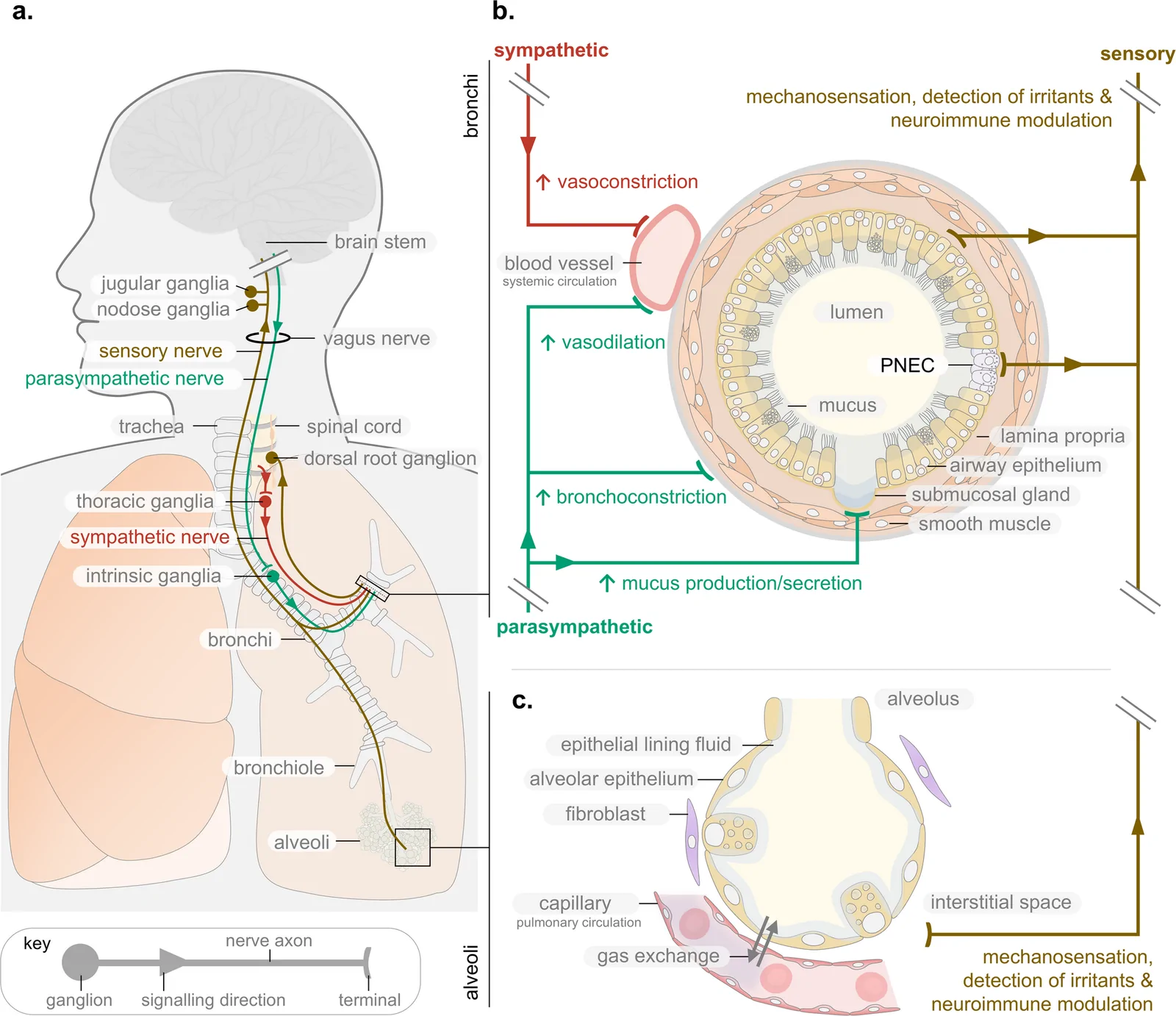
Compartment- and nerve-type-specific peripheral innervation of the lung. Schematic overview of peripheral nervous system (PNS) pathways regulating lung physiology across airways, vasculature and alveolar regions. **(a)** Autonomic efferent and sensory afferent pathways innervating the lung. Preganglionic parasympathetic neurons arise in the brainstem and project through the vagus nerve to intrinsic parasympathetic ganglia located within or adjacent to the tracheobronchial wall. Postganglionic cholinergic fibres subsequently innervate airway smooth muscle, the secretory epithelium and submucosal glands. Sympathetic preganglionic neurons arise in the thoracic spinal cord and synapse within thoracic sympathetic chain ganglia, from which postganglionic fibres project predominantly to pulmonary and bronchial blood vessels. **(b)** Neural regulation and sensory surveillance of a cartilaginous bronchus. The bronchial wall comprises a mucus-covered, ciliated pseudostratified epithelium containing rare pulmonary neuroendocrine cells (PNECs), which may occur individually or in clusters termed neuroepithelial bodies, overlying the lamina propria, airway smooth-muscle bundles, submucosal glands and cartilage. PNECs function as innervated epithelial sensors that detect environmental, chemical and mechanical stimuli and release neurotransmitters or neuropeptides that may influence sensory neurons and local immune cells. Parasympathetic signalling induces muscarinic receptor-mediated airway smooth muscle contraction and stimulates glandular mucus secretion. Sympathetic nerves can induce vasoconstriction of bronchial vasculature. Sensory afferents within the airway epithelium detect mechanical changes, inhaled irritants, inflammatory mediators (immunomodulation) and other chemical stimuli, initiating protective respiratory and autonomic reflexes. **(c)** Innervation within the alveolar compartment. The alveolar surface is lined by a surfactant-containing epithelial fluid layer and is composed of thin type I alveolar epithelial cells and surfactant-producing type II alveolar epithelial cells. Pulmonary fibroblasts reside within the interstitial space, and pulmonary capillaries are in close proximity to the epithelium, facilitating efficient gas exchange. Sensory nerve endings located within the alveolar interstitium and near the pulmonary capillary endothelium monitor mechanical and chemical changes associated with ventilation and gas exchange, detect irritants, and contribute to the regulation of immune responses. *Green denotes parasympathetic efferent pathways*,* red denotes sympathetic efferent pathways*,* and brown denotes vagal or spinal sensory afferent pathways. Ganglia*,* axons*,* terminals and anatomical distances are shown schematically and are not drawn to scale. Figure created by the author*,* using presentation graphics software*

In the lung, physiological processes regulated by the PNS are coordinated in a nerve-type- and compartment-specific manner. These influences include a network of sensory fibres that detect local chemical and mechanical stimuli, and autonomic efferents that regulate airway smooth muscle tone, mucosal secretion and pulmonary vascular responses (e.g., regulation of blood flow) through parasympathetic and sympathetic pathways [2, 6–9] (Fig. 1; Table 1). Pulmonary nerves orchestrate their functions and responses through a diverse range of signalling molecules, including neurotrophins, neuronal guidance proteins and neurotransmitters. Neurotrophins are proteins that promote neuronal growth and survival and play key roles in establishing and maintaining lung innervation, and include nerve growth factor (NGF), brain-derived neurotrophic factor (BDNF), neurotrophin-3 (NT-3) and neurotrophin-4 (NT-4) [39–41]. During lung development and tissue repair, the growth and patterning of nerve fibres are directed by families of neuronal guidance proteins, which provide molecular guidance cues for neurites and include netrins (netrin-1, -3 and -4), ephrins (ephrin-A and -B), semaphorins (classes 3, 4 and 7), and slits (slit-1, -2, and - 3) [42–46]. Neurotransmitters are broadly classified into small molecules or neuropeptides. Small-molecule transmitters include acetylcholine, noradrenaline, and dopamine, whereas neuropeptides are larger peptide messengers, including substance P (SP), calcitonin gene-related peptide (CGRP) and neuromedin U (NMU). Released from sensory and autonomic nerve terminals in response to physiological or environmental stimuli, these mediators regulate airway smooth muscle tone, vascular tone, mucus secretion and local immune activity [22, 23]. Thus, neuronal guidance proteins and neurotrophins establish and refine the lung’s neural architecture, while neurotransmitters serve as rapid chemical signals that mediate its physiological functions (Table 1).

As a barrier organ, the lung is continuously exposed to airborne pathogens, allergens, and environmental pollutants, so the pulmonary nerves interact with other cells to facilitate responses to environmental and inflammatory/immune cues. This process occurs, in part, through the release of immunomodulatory mediators that regulate local immune activity. This neuro-immune crosstalk enables a coordinated host defence and subsequent tissue repair. However, when this crosstalk is dysregulated, it can contribute to a broad spectrum of pulmonary diseases characterised by chronic inflammation, airway hyperresponsiveness and pathological tissue remodelling [10–12, 47, 48]. In addition to immune cells, specialised epithelial populations also participate in pulmonary neurobiology. Pulmonary neuroendocrine cells (PNECs) are rare airway epithelial cells, constituting 0.01–0.5% of the airway epithelium [49], found individually or within sensory-innervated neuroepithelial bodies. Acting as local sensory and secretory nerve-lung hubs, they respond to hypoxia, mechanical stress and inhaled stimuli by releasing mediators such as serotonin, gamma-aminobutyric acid (GABA) and CGRP, thereby influencing airway tone, mucus secretion, vascular responses and inflammation [50].

**Table 1:** Neurotransmitter signalling pathways in pulmonary innervation. Summary of major sensory, parasympathetic, and sympathetic nerve types in the airways, including their primary pulmonary functions, principal neurotransmitters, cognate receptors, and downstream functional effects on bronchial tone, mucus secretion, vascular regulation, immune and fibroblast modulation. Afferent (signal towards CNS) and Efferent (signal from CNS)

Nerve Type		Description	Neurotransmitter Signalling			Ref.
			Neurotransmitter	Receptor	Functional Output	
Afferent	Sensory	Pulmonary sensory afferents arise predominantly from the vagal nodose and jugular ganglia, with a smaller contribution from spinal dorsal root ganglia. Myelinated A-fibres rapidly detect mechanical changes, including lung inflation and deflation, whereas TRPV1- and TRPA1-expressing unmyelinated C-fibres sense irritants, allergens, pathogens and inflammatory signals. Activation of TRPV1-expressing nociceptors can release substance P and calcitonin gene-related peptide from peripheral terminals, thereby modulating vascular permeability, mucus secretion, and immune cell recruitment. Acting as local sensory-secretory hubs in the airway, pulmonary neuroendocrine cells respond to noxious stimuli by releasing neurotransmitters to activate adjacent vagal sensory afferents.	substance P (SP)	neurokinin-1 receptor (NK1R) and neurokinin-2 receptor (NK2R)	bronchoconstriction, mucus secretion, dendritic cell signalling	[5, 8, 10–21]
			calcitonin gene-related peptide (CGRP)	calcitonin receptor-like receptor (CRLR)/receptor activity-modifying protein 1 (RAMP1) heterodimer	vasodilation, immunomodulation (lymphocyte and dendritic cells)	
			vasoactive intestinal peptide (VIP)	vasoactive intestinal polypeptide receptor 1 (VPAC1) and 2 (VPAC2)	bronchodilation, homeostatic mucus secretion, alveolar macrophage signalling	
			neuromedin U (NMU)	neuromedin U receptor 1 (NMUR1)	Inflammation response Group 2 innate lymphoid cells (ILC2s)	
Efferent	Parasympathetic	The parasympathetic nerve’s primary function is to promote bronchoconstriction and mucus secretion during homeostatic or defensive reflexes, limiting airflow and protecting the distal parenchyma from inhaled irritants. Parasympathetic pulmonary innervation arises from medullary brainstem neurons that travel via the vagus nerve to synapse in parasympathetic ganglia embedded within the airway wall. Acetylcholine released from parasympathetic fibres acts on muscarinic receptors to drive bronchoconstriction, mucus secretion and airway remodelling, while presynaptic acetylcholine receptor type 2 limits further acetylcholine release. Non-adrenergic, non-cholinergic parasympathetic fibres also release vasoactive intestinal peptide, promoting vasodilation, bronchodilation and context-dependent modulation of immunity.	acetylcholine (ACh)	muscarinic acetylcholine receptor type 1 (mAChR1 or M1) muscarinic acetylcholine receptor type 2 (mAChR2 or M2) muscarinic acetylcholine receptor type 3 (mAChR3 or M3)	mucus secretion ACh inhibitory autocrine feedback bronchoconstriction, mucus secretion, vasodilation (endothelial)	[22–26]
			vasoactive intestinal peptide (VIP)	vasoactive intestinal polypeptide receptor 1 (VPAC1) and 2 (VPAC2)	vasodilation, bronchodilation, immunomodulation	
	Sympathetic	Their primary function is to promote bronchodilation and modulate mucosal vascular tone during systemic fight-or-flight responses, increasing airflow. Sympathetic pulmonary innervation arises from preganglionic neurons within the thoracic spinal cord that synapse in the paravertebral sympathetic chain, subsequently projecting postganglionic fibres to the lungs, and releasing mainly noradrenaline. In humans, these fibres are concentrated around the bronchial vasculature, where they regulate vascular tone, whereas direct innervation of airway smooth muscle is relatively sparse. They have also been reported to modulate immune responses and fibroblast biology Sympathetic innervation is predominantly dopaminergic early in life but shifts to a noradrenergic phenotype in adulthood. They also co-release neuropeptide Y with noradrenaline, which has vasoconstrictive and immunomodulatory properties.	Noradrenaline (NA) (also known as norepinephrine (NE))	β_2_-adrenoceptor α_1_-adrenoceptor activation	bronchodilation (primarily via circulating adrenaline), reduces pulmonary secretions vasoconstriction (via smooth muscle) immunomodulation and fibroblast activation	[6, 22, 23, 27–38]
			Dopamine	D_1_-like receptors D_2_-like receptors	bronchodilation, immunomodulation vasodilation fibroblast modulation	
			Neuropeptide Y (NPY)	Y Receptor (1, 2, 4 and 5)	vasoconstriction, immunomodulation	

### Pulmonary Neuropathology

Peripheral nerves are increasingly recognised as active regulators of both tissue homeostasis and disease pathogenesis across multiple organ systems. Pathological changes in peripheral tissue nerve density, hyperinnervation, or loss of nerve density are linked to impaired regeneration in the skin [51] and heart [52], age-related thymic involution [53], vascular senescence and cardiac dysfunction [54], obesity-associated adipose tissue dysfunction [55], and cancer pathogenesis [56, 57]. In the lung, the peripheral nerves have been implicated in a broad range of respiratory functions. Consequently, pathological remodelling of pulmonary nerves and dysregulation of neurotransmitters may contribute to a broad range of conditions including acute inflammation in lung injury, airway hyperreactivity in asthma, alveolar remodelling in pulmonary fibrosis, and tumour progression in lung cancer [4, 10, 58, 59].

## Acute Lung Injury, Infection and Inflammation

Acute lung injury (ALI) is an inflammatory syndrome characterised by rapid disruption of the alveolar-capillary barrier, pulmonary oedema, impaired gas exchange, and in severe cases progression to acute respiratory distress syndrome (ARDS) [60]. In animal models of ALI, pulmonary **sensory neurons** can detect tissue damage signals and pro-inflammatory cytokines via the transient receptor potential vanilloid 1 (TRPV1) channel [12, 61], and release neuropeptides including SP, CGRP and VIP, which contribute to both pro- [61–63] and anti-inflammatory [64–66] responses. For instance, mouse modelling has shown that sensory nerves can enhance antiviral protection, but there is also evidence that they can reduce antibacterial defence [10, 11, 67]. Specifically, in a murine model of influenza A virus infection, chemical and genetic ablation of TRPV1^+^ sensory neurons promotes protective myeloid interferon signalling, thereby reducing the accumulation of pathogenic (*Nmes1*^+^) neutrophils [14]. On the other hand, TRPV1^+^ sensory neurons also suppress protective innate immunity in a murine model of acute *Staphylococcus aureus* pneumonia via the local release of CGRP, which limits neutrophil recruitment and cytokine responses [15]. Sensory nerves can also induce a systemic response to injury, characterised using rodent models of ALI, by sending afferent (injury) signals to the brainstem, which in turn (i) signals the efferent cholinergic anti-inflammatory pathway to the splenic nerve where (ii) noradrenaline is released in the spleen, activating β2-adrenergic receptors on a subset of memory T lymphocytes expressing choline acetyltransferase (ChAT), (iii) inducing acetylcholine production and (iv) systemic inhibition of macrophage TNF-α release [68–70].

Autonomic signalling also modulates acute lung injury through receptor- and cell-specific mechanisms. In lipopolysaccharide (LPS)-induced ALI, and LPS exposure to human endothelial cell culture, **parasympathetic** acetylcholine signalling via the muscarinic acetylcholine receptor type 3 (mAChR3) is linked to exacerbation of pulmonary microvascular endothelial injury and vascular hyperpermeability [71, 72]. In contrast, **sympathetic** dopaminergic signalling induces antioxidant programmes, suppresses macrophage-driven inflammation and supports epithelial tight-junction integrity [73], and noradrenergic signalling exhibits context-dependent effects. In a murine model of *Escherichia coli* pneumonia, sympathetic amplification can worsen injury by stimulating a β2-adrenoceptor-positive interstitial macrophage subset and exacerbating pro-inflammatory cytokine release [74], though in murine LPS-induced ALI, β2-adrenergic signalling has demonstrated anti-inflammatory roles, in other pulmonary macrophage populations [75, 76]. These opposing functions may explain the lack of clinical benefit observed with β2-adrenoceptor agonists in ALI clinical trials [77, 78]. Further study of the interplay between sensory innervation and autonomic nervous system in human relevant injury models could yield new mechanistic insights with therapeutic impact.

## Asthma

Asthma is a chronic inflammatory airway disease, characterised by airway hyperresponsiveness, bronchoconstriction, and mucus hypersecretion that converge on airway narrowing and airflow restriction [79, 80]. In type 2-high (allergic) asthma, disease mechanisms are predominantly mediated by T-helper type 2 (Th2) cells, group 2 innate lymphoid cells (ILC2s), eosinophils, and mast cells, while type 2-low (non-allergic) asthma is associated with neutrophil inflammatory responses or other non-inflammatory processes [79]. Although immune and epithelial pathways are extensively characterised in asthma pathogenesis, they function within a densely innervated airway microenvironment subject to neural regulation. Sensory and autonomic nerves can both detect inflammatory cues and actively modulate immune responses, highlighting their potential to contribute to inflammatory conditions, including asthma [2, 81].

A well-characterised **sensory** nerve example involves the neuropeptide NMU in type 2-high (allergic) asthma, which acts on key effector cells, including ILC2s and eosinophils. Following airway allergen exposure, sensory nerves release NMU, which binds to its receptor NMUR1, a receptor that is highly expressed on lung-resident ILC2s in both animal models and humans with asthma, and on eosinophils, resulting in the release of pro-inflammatory cytokines [82–84]. ILC2s may also be activated by the PNECs, which are innervated by sensory neurons and increasingly recognised in asthma pathogenesis [85].

Given that **parasympathetic** nerves regulate bronchoconstriction via acetylcholine-mediated activation of mAChR3 receptors on airway smooth muscle cells, dysregulation of this pathway can exacerbate airway narrowing. Under normal conditions, inhibitory neuronal mAChR2 autoreceptors provide negative feedback to limit acetylcholine release from parasympathetic nerves. In asthma, however, eosinophils recruited to the airway release major basic protein, which inhibits neuronal mAChR2 receptors and disrupts this feedback mechanism [86, 87]. In murine models of house dust mite-induced asthma, mast cell-derived serotonin interacts with nerve 5-HT2A receptors to induce further release of parasympathetic nerve-derived acetylcholine. Supported in vitro by the identification of functional mAChR3 on primary human lung mast cells, this mechanism creates a positive feedback loop resulting in excessive acetylcholine signalling and increased bronchoconstriction [88, 89]. Notably, elevated acetylcholine levels in asthmatic and other inflamed airways are not solely of neuronal origin but also arise from the non-neuronal cholinergic system (NNCS), with emerging evidence implicating neutrophils as a significant source [90, 91]. Chronic allergen exposure and inflammation can also drive pathological remodelling of parasympathetic nerves in asthma. Neurotrophins, particularly BDNF, promote nerve branching and local hyperinnervation, increasing nerve terminal density and acetylcholine release, which in turn, enhances pathological bronchoconstriction and mucus secretion [92, 93]. NGF, another key neurotrophin, may also contribute to hyperinnervation, as it is elevated in the bronchoalveolar lavage fluid (which predominantly captures components of the epithelial lining fluid, Fig. 1c) and bronchial biopsy specimens of asthma patients [94, 95], and increases in animal models of allergen challenge [96].

Despite the knowledge of neuronal contribution to asthma, studies into the pathomechanisms of asthma have predominantly focused on the airway epithelium, smooth muscle and the immune system, reflecting their central roles in the disease pathogenesis [79, 80, 97]. Accordingly, contemporary and emerging asthma therapeutics principally target the epithelial-immune-smooth muscle axis [98, 99], with neural targets remaining underrepresented as direct therapeutic targets and highlighting an important potential area for future investigations.

## Pulmonary Fibrosis

Pulmonary fibrosis encompasses a group of interstitial lung diseases characterised by excessive extracellular matrix deposition and progressive tissue remodelling in response to recurrent epithelial injury and aberrant fibroblast proliferation and differentiation. Clinically, it presents with cough, dyspnoea, gas exchange impairment, and frequently progresses to respiratory failure and death [100].

In pulmonary fibrosis, **sensory** nerves are currently viewed as anti-fibrotic [4]. For example, CGRP has been reported to exert broad anti-fibrotic effects by preserving alveolar epithelial integrity, restraining fibroblast activation, and limiting pro-fibrotic macrophage polarisation. Evidence from CGRP-deficient mouse models demonstrates that endogenous CGRP protects alveolar type 2 cells from inflammatory senescence, oxidative stress and apoptosis in a systemic ageing model [101, 102]. CGRP deficiency is also associated with enhanced pro-fibrotic macrophage polarisation and increased collagen deposition in the bleomycin-induced lung fibrosis model [101]. In vitro studies on rodent primary pulmonary fibroblasts show that exogenous CGRP, as well as VIP, can suppress TGF-β1-induced fibroblast activation [103, 104].

Autonomic pathways regulate pulmonary fibrosis through transmitter- and receptor-specific mechanisms [6]. Within the lung, **parasympathetic** acetylcholine signalling may act as a pro-fibrotic amplifier. In vitro, pharmacological activation of the α7 nicotinic acetylcholine receptor (α7 nAChR), on primary lung fibroblasts, upregulates the expression of key fibrogenic genes (*Acta2*; fibroblast-to-myofibroblast differentiation, and *Col1a1*; collagen expression) [105]. Additionally, in vitro data from primary human lung fibroblasts demonstrated that mAChR3 activation induced fibroblast proliferation, which can be attenuated by the anti-muscarinic agent tiotropium bromide [106]. In vivo data are limited. Earlier evidence suggests that vagotomy (the severing of vagus nerve branches) in murine bleomycin-induced lung fibrosis models attenuates fibrotic outcomes [107]; however, a recent report of a rat lung vagotomy showed increased fibrotic readouts [108], highlighting potential species- or context-dependent effects.

**Sympathetic** signalling is emerging as a key mediator in pulmonary fibrosis, with evidence supporting a pro-fibrotic role for noradrenaline and an anti-fibrotic effect of dopamine. In both human pulmonary fibrosis tissue and mouse bleomycin-induced fibrosis, local noradrenaline levels are increased. Macrophage-derived netrin-1 is a key neuronal guidance cue that promotes sympathetic nerve remodelling within the distal lung parenchyma, and conditional deletion of myeloid *Ntn1* prevents this aberrant innervation, thereby limiting presynaptic terminal accumulation and reducing noradrenaline. Additionally, α1-adrenoreceptor antagonism (using terazosin) decreases bleomycin-induced collagen deposition and improves histological readouts, potentially acting through α1-adrenoreceptor-expressing immune cells and fibroblasts [37]. This pro-fibrotic adrenergic effect in pulmonary fibrosis was further characterised in a recent publication, which utilised (i) whole-lung optical clearing with 3D light-sheet microscopy to map aberrant sympathetic innervation in mouse bleomycin-induced lung fibrosis, (ii) genetic sympathetic nerve ablation, myofibroblast-specific *Adra1d* deletion and human pulmonary fibrosis samples to define a pathogenic nerve-fibroblast axis, as well as in vitro approaches, (iii) where sympathetic nerve co-culture with pulmonary fibroblasts amplified transforming growth factor-β1 (TGF-β1)-induced fibroblast differentiation, and (iv) terazosin treatment of human precision-cut lung slices (hPCLS) attenuated fibrotic-cocktail-induced fibrotic readouts [36]. Adrenergic nerves have also been implicated in a murine model of sepsis-associated pulmonary fibrosis, in which increased noradrenaline and pulmonary fibroblast differentiation were attenuated by α2A-adrenoreceptor antagonist (yohimbine), and further exacerbated by α2A-adrenoreceptor agonist (clonidine) [109]. Conversely, sympathetic dopaminergic signalling exerts anti-fibrotic effects. A recent report found that dopamine receptors D1 (D1R) and D2 (D2R) are upregulated in myofibroblasts in both IPF patient lungs and bleomycin-induced mouse models, and activation of these receptors suppresses TGF-β1-driven myofibroblast differentiation, reducing extracellular matrix deposition and fibrosis progression in vivo. Consistently, selective agonists for D1R and D2R attenuate bleomycin-induced pulmonary fibrosis by limiting fibroblast proliferation and differentiation [38]. Interestingly, unlike the β2-adrenergic receptor on lung fibroblasts, which is downregulated following TGF-β exposure, D1R expression is preserved within the context of a pro-fibrotic (TGF-β-high) microenvironment, potentially allowing for dopamine to provide sustained anti-fibrotic signalling [110]. Understanding innervation in human-relevant models of pulmonary fibrosis, particularly interactions across stromal- and immune-neural axes, may uncover novel mechanisms of disease progression and identify therapeutically actionable pathways.

## Lung Cancer

Lung cancers are classified based on their cellular origin and are broadly divided into two main categories. Small-cell lung cancer (SCLC) is an aggressive, rapidly metastasising malignancy of neuroendocrine origin, whereas non-small-cell lung cancer (NSCLC) is a heterogeneous group of carcinomas originating from pulmonary epithelial cells (comprising primarily adenocarcinomas and squamous cell carcinomas) [111]. Neuronal signalling is increasingly recognised as an active component of the tumour microenvironment [57, 59, 112, 113], and may actively recruit nerves through the release of neurotrophins and neuronal guidance proteins [114–116].

Broadly, **sensory** nerves can have opposing effects on cancer development, either promoting or suppressing tumour growth, depending on where nerve-immune interactions occur within the tissue, the types of sensory nerve fibres present, and how nerve injury changes over time [13, 117, 118]. In the lung, sensory nerves may contribute to worsening carcinogenesis. In recent publications, using in vivo murine lung adenocarcinoma models, it was reported that tumour-associated sensory networks facilitate immune evasion, through macrophage-mediated pathways, via a systemic vagal-sympathetic interoceptive circuit or through local nociceptive paracrine signalling, promoting lung adenocarcinoma progression by suppressing anti-tumour immunity [119, 120]. Consistently, in vitro co-culture of human and mouse SCLC cell lines with glutamatergic neurons increases tumour cell proliferation, an effect that is reduced by tetrodotoxin, a blocker of voltage-gated sodium channels that inhibits neuronal action potentials [121].

The **parasympathetic** nervous system can also exert opposing effects on carcinogenesis, acting as either a tumour suppressor or a driver [122]. In the lung, the role of parasympathetic nerves in cancer biology is complicated, given that non-neuronal cells, including lung tumour and immune cells, can also produce acetylcholine [123]. This finding makes it difficult to attribute this signalling to neurons alone, but still highlights the important role of the neurotransmitter in carcinogenesis. Nevertheless, pharmacological inhibition of mAChR2 or mAChR3 can inhibit tumour growth in vivo, potentially by suppressing epithelial-mesenchymal transition (EMT), which would otherwise drive the transcriptional reprogramming that enables epithelial tumour cells to acquire invasive, metastatic phenotypes [124–126].

**Sympathetic** adrenergic signalling has a strong pro-tumorigenic profile [116]. This was demonstrated in a recent study, where adrenergic signalling was associated with multiple anti-tumorigenic effects. Using a mouse model of lung cancer, they showed that depletion of endogenous catecholamines with 6-hydroxydopamine hydrobromide (6-OHDA) reduces intra-tumoral catecholamine levels, suppresses M2-like (pro-tumorigenic) macrophage activity, attenuates tumour neovascularisation, and inhibits tumour growth. Further, in vitro catecholamine exposure promotes macrophage M2-like polarisation and increases VEGF expression, effects that are reversed by the adrenergic receptor antagonist propranolol [127]. Consistent with these findings, subsequent studies have also shown that noradrenaline signalling in lung cancer cells promoted proliferation, invasion and metastatic potential in vivo, and pharmacological inhibition of β2-adrenoreceptor or surgical sympathectomy (severing of sympathetic nerves) reduced tumour growth and prolonged survival [128]. Clinical evidence from cancer patients taking β-blockers for non-malignant indications reveals improved overall and recurrence-free survival, as well as a reduction in metastasis occurrence, highlighting the translational potential of targeting neurotransmitter signalling in disease [129].

### Translational Experimental Models in Pulmonary Neurobiology

In recent years, research into pulmonary pathophysiology has largely centred on epithelial, mesenchymal, immune and vascular compartments, partially reflected by the methodological challenges associated with studying peripheral nerves. Specifically, neuronal cell bodies are located outside the lung and exert their influence within the tissue through sparse, fragile terminal networks. Consequently, these organ neuronal structures are difficult to preserve, isolate, and analyse using conventional methods. For instance, conventional human lung transcriptomic approaches, including single-cell and single-nucleus RNA sequencing (scRNA-seq, snRNA-seq), have transformed our understanding of pulmonary pathophysiology, providing high-resolution reference maps (atlases) that enable the discovery of rare cell populations, delineation of developmental and disease-associated cell states [49, 130], yet neuronal populations are absent in these datasets. This limitation reflects the location of sensory and autonomic neuronal cell bodies (somata) outside of the lung, in extrapulmonary ganglia. Consequently, neither standard scRNA-seq, which requires viable whole-cell dissociation, nor snRNA-seq, which requires cell nuclei, can fully capture neuronal transcriptomes from lung tissue. While spatial transcriptomics can map nerves within the pulmonary architecture, axonal and terminal RNA is low in abundance [131, 132], so high-resolution platforms, such as Visium HD, may improve detection sensitivity [133]. Other methodologies, including immunofluorescence, can also be challenging due to the scarcity of nerves and the fine calibre of individual neurites [134]. For the same reasons, ex vivo modelling using hPCLS, although preserving native lung architecture, including some sparse axons and nerve terminals, is limited because these fibres are severed from their neuronal somata and have limited functional viability [135, 136], thereby decoupling the pulmonary central reflex circuits and some dynamic processes, such as innervation. With these challenges, the neurobiological dimension of human pulmonary pathophysiology remains comparatively underexplored and highlights the importance of employing robust experimental frameworks to model lung-nerve biology with anatomical, cellular and functional fidelity.

Rodent models are indispensable for defining biological mechanisms within intact neural circuits and establishing how peripheral sensory and autonomic pathways influence pulmonary pathophysiology. Cell-specific genetic approaches enable the study of interactions between nerves and non-neuronal compartments, including relevant effector cell populations such as PNECs, through depletion [137], and pulmonary mesenchymal populations via conditional gene excision of neurotransmitter receptors [36] and of neural guidance proteins [37]. Meanwhile, optogenetic approaches enable nerve-specific modulation by genetically inserting light-sensitive ion channels into specific neurons [138, 139], and chemogenetic approaches, which use genetically engineered receptors that are unresponsive to endogenous ligands, can selectively activate or inhibit neurons by using small molecules [119, 140, 141]. Further nerve-specific modulation can be achieved through selective denervation strategies, including chemical [36, 142] and genetic ablation [142], enabling functional evaluation of neural contributions to disease. Notably, the whole-organism context also permits transcriptomic profiling of peripheral ganglia [143], providing insight into how neurons respond to lung-derived signals. Rodent models also provide a platform for studying how neural circuits influence lung disease within intact three-dimensional networks, in which advanced lung imaging methods, such as confocal microscopy and optical clearing with light-sheet microscopy, have been used to map innervation patterns. This approach is particularly valuable given the lung’s complex three-dimensional structure and the highly branched nature of pulmonary nerves, which are difficult to fully capture in two-dimensional sections during homeostasis [134, 144], ALI [32], pulmonary fibrosis [36], and lung cancer [119, 144]. While rodent models remain essential for mechanistic discovery, species-specific differences, including in receptor and ion-channel expression, neuronal subtype composition and regional innervation [2, 145–147] highlight the need to complement these models with robust human in vitro systems that enable validation in a clinically relevant cellular context.

Primary human peripheral neurons are difficult to study in culture settings because they are post-mitotic, making them both challenging to isolate and unable to be readily expanded due to their lack of proliferative capacity [148]. Instead, approaches to generate human peripheral neurons in vitro utilise cancer- or stem cell-derived lines, which enable scalable expansion followed by directed differentiation into functionally relevant neuron-like cells, including immortalised cell lines and pluripotent stem cells (PSCs). Cancer-derived neuronal cell lines are tractable, scalable models of human neuronal biology, with SH-SY5Y among the most widely used [149, 150]. This human neuroblastoma line, derived from SK-N-SH, can be differentiated into cholinergic- or catecholaminergic neuron-like states, including dopaminergic [151, 152] and adrenergic phenotypes [153, 154]. Its neural crest origin supports responsiveness to differentiation cues and retention of catecholamine machinery, allowing for relevant mechanistic studies and high-throughput screening [150, 155], as well as more advanced approaches, including 3D- and co-culture systems [156–158]. Their tumourigenic origin, altered metabolic state and intrinsic phenotypic immaturity necessitate rigorous differentiation and validation to approximate a mature, homogeneous neuronal phenotype [149, 150].

Human pluripotent stem cells (hPSCs), including human embryonic stem cells (hESCs) and human induced pluripotent stem cells (hiPSCs), have the capacity to self-renew indefinitely and differentiate into all somatic cell types, providing a scalable platform for disease modelling, developmental studies and regenerative medicine. hiPSC lines can retain residual epigenetic memory of their donor cells, potentially contributing to inter-line differences in differentiation propensity, and differentiated hPSCs often remain developmentally immature or foetal-like and therefore only partially recapitulate adult phenotype and function. Therefore, many differentiation protocols use transcriptomic, proteomic and functional validation to approximate target cell identity and maturity [159–161]. hPSCs can be differentiated using defined combinations of growth factors and small molecules to direct lineage specification, or through viral vector-mediated transcription factor overexpression. Chemically defined differentiation protocols offer advantages over viral approaches, as they preserve genomic integrity by avoiding insertional mutagenesis and enable precise, reproducible modulation of endogenous signalling pathways. By applying defined combinations of growth factors and small-molecule inhibitors in a stage-specific sequence, hPSCs can be guided through developmental intermediates that reflect embryogenesis, leveraging developmental biology to direct cell fate [160]. Accordingly, differentiation protocols for peripheral sensory and autonomic neurons broadly recapitulate developmental trajectories. **Sensory** neurons originate from Sox10/FoxD3-positive trunk neural crest cells, and their specification is governed by Neurog1/2, followed by Pou4f1-driven differentiation, generating subtypes marked by Runx1/Runx3 and TrkA/B/C expression [162, 163]. **Parasympathetic** neurons arise from Sox10-positive precursors, which undergo Phox2b- and Ascl1-dependent neurogenesis with loss of Sox10 and acquisition of TUBB3 and peripherin [164]. **Sympathetic** neurons derive from Sox10-positive trunk neural crest cells, and following BMP2/4/7 signals, they activate Ascl1-, Phox2a/b-, and Hand2-dependent programmes, forming TH/DBH-positive sympathoadrenal progenitors that mature into sympathetic neurons [165]. Consistent with the growing recognition of neural regulation as a critical component of lung biology and disease, and alongside rapid advances in hPSC differentiation methodologies, the field is now poised at an exciting point of convergence. Although applications of hPSC-derived peripheral neurons in pulmonary research remain relatively limited, their expanding use in other systems highlights clear opportunities for translation, positioning these models as an emerging platform to investigate nerve-lung interactions (Table 2). For example, **sensory** neuron protocols generating nociceptors could model neuro-immune signalling in airway hypersensitivity [167], while those producing mechanoreceptive neurons may be suited to studying lung stretch reflexes and mechanical stress in conditions such as pulmonary fibrosis [168]. **Parasympathetic** neuron models have been successfully used in functional co-cultures with human airway smooth muscle cells, supporting relevance to asthma [170], with other relevant examples in genetic diseases, viral infections [171], and co-culture systems with cardiomyocytes [173]. **Sympathetic** neuron models have been applied in organoids and co-cultures with cardiac and endocrine tissues with methods that could be extended to examine sympathetic regulation of pulmonary circulation and airway blood flow [175, 178]. Importantly, in the absence of primary cell comparators, SH-SY5Y cells offer an accessible, baseline in vitro comparator to support the validation of hPSC-derived neuron-related discoveries [179–182]. Collectively, these sensory and autonomic differentiation studies demonstrate the suitability of PSC-derived neurons for understanding cell-cell crosstalk and provide an important technical foundation for more complex multicellular models.

**Table 2:** Methods for generating and characterising hPSC-derived sensory, parasympathetic, and sympathetic neurons. Summary of published approaches used to differentiate human pluripotent stem cells (hPSCs), including human embryonic stem cells (hESCs) and human induced pluripotent stem cells (hiPSCs), into peripheral neuronal subtypes relevant to airway innervation, including nociceptive sensory, parasympathetic, and sympathetic neurons. For each protocol, the table outlines the differentiation strategy, key developmental or mature neuronal markers assessed at the transcriptional level, protein-level validation methods, and functional assays used to confirm neuronal identity and activity. This table focuses on methods that modulate endogenous signalling pathways without direct genetic manipulation, and therefore does not include viral or transgene-driven approaches

Nerve Type	hPSC-Derived Nerves	Characterisation		
		Transcriptional	Protein-Level	Functional
Sensory	**Nociceptive sensory neurons** [166] hESCs and hiPSCs are treated with a combination of three small molecules SU5402, CHIR99021, and DAPT, to give rise to sensory neurons at day 12. Forms the foundation for many of the current sensory neuron protocols. Considerations: limited characterisation of contaminating cell types, and only gives rise to nociceptive sensory neurons (not mechanoreceptive or proprioceptive)	**Microarray based transcriptomics to map the developmental kinetics:** (*OCT4, DLK1, PAX6, SOX10, POU4F1 (BRN3A), ISL1, NEUROG2, NEUROG1, NTRK1, VGLUT2, TAC1 and TRPV1*), neuronal stage expression of mature nociceptive markers (*SCN9A, SCN10A, SCN11A, P2RX3, TPRV1, and TRPM8*)	**FACS:** at neuron stage (NTRK1+) **Immunofluorescence:** at neural crest stage (SOX10) at neuron stage (ISL1, BRN3A, TUJ1, Glutamate, RUNX1, RET, PRPH)	patch-clamp experiments live cell calcium imaging
	**Nociceptive sensory neurons** [167] hESCs and hiPSCs are first differentiated into neural crest, maintained as nocispheres, and finally terminally differentiated into sensory neurons. Considerations: automated hPSC cell culture may be impractical for many laboratories	**Bulk RNA-seq:** at pluripotency (*POU5F1 and NANOG*) at neural ectoderm (*SOX2, SOX21, OTX2*) at neural crest (*PAX3, NEUROG1, SOX10, TFAP2A, TFAP2B*) at neuron stage (*POU4F1, ISL1, DRGX, SLC17A6, NTRK2, SCN9A, SCN10A, SCN11A, RUNX1, TRPV1, NRG2, BDNF, NGF, including mature functional markers P2RX3, TRPV1, OPRL1, C1, CALCA*) **RT-qPCR:** at neuron stage (*P2RX3, TRPV1, TAC1*) **In situ hybridisation:** (NaV1.7, 1.8, and 1.9 mRNA expression and co-localisation at the cellular level)	**Immunofluorescence:** (TUJ1, BRN3A, SOX10, PRPH, ISL1, VGLUT1, MAP2, NF200, Tau at neural crest and neuron stages)	Multielectrode Array/MEA live cell calcium imaging patch-clamp experiments
	**Nociceptive, proprioceptive, and mechanoreceptive sensory neurons** [168] hESCs and hiPSCs are first differentiated into neural crest cells, and replated either on day 6 or 12 to terminally differentiate into mechanoreceptive or sensory neurons with a DRG-like composition, respectively. Considerations: immunopanning technique may be too complex/impractical for many laboratories	**RT-qPCR:** at neuron stage: (*BRN3A, ISL1, NGN2, RUNX3, TRKC, RET, TRKB, NGN1, RUNX1, TRKA*) nociceptor markers (*SST, TAC1, TRPV1, TRPV2, SCN8A, SCN9A, SCN10A, SCN11A, PLXNC1, TRPM8, TRPA1, P2X3*) proprioceptors markers (*SPP1, PVALB*) mechanoreceptor markers (*KCNK2, KCNK4, ASCI1, ASCI2, ASCI3, PIEZO2, NF200, NECAB2, FAM19A1*)	**FACS:** at neural crest stage (CD49d+) **Immunofluorescence:** at neural crest stage (SOX10, AP2a, BRN3A) at neuron stage (ISL1, TUJ1, RUNX1, RET, PRPH, BRN3A, VGLUT1, VGLUT3, SubP, CGRP, TRKA, TRKB, TRKC)	Multielectrode Array/MEA
	**Sensory neurons** [169] hESCs derived caudal neural progenitors are induced, purified towards SOX10+ neural crest neurospheres, then terminally differentiated to sensory neurons. Considerations: lower efficiency of SOX10+ neural crest (~60%), and the sensory neuron protocol requires floating spheroid cultures and multiple replating steps	**RT-qPCR:** at neurosphere stage (*SOX10, BRN3A, RUNX1, RUNX3*) at neuron stage (*PRPH, RET, TRKA, TRKB, TRKC*)	**Immunofluorescence:** at neuron stage (BRN3A, ISLET1, NF200, TRKB, TRKC, NECAB2, TRKC, FAM19A1, SPP1, PV, TRKA, TRPV1, PLXNC1, SST, NK-1)	Multielectrode Array/MEA
Parasympathetic	**Peripheral cholinergic neurons** [170] hESCs are differentiated into vagal neural crest precursors, maintained in a 3D spheroid stage, and terminally differentiated to cholinergic neurons. Considerations: relatively immature phenotype compared to primary cells, and limited spontaneous firing activity	**RT-qPCR:** at hPSC stage (*NANOG, OCT4*) at neural crest stage (*PAX3, HOXB3, HOXB5*) at neuron stage (*ASCL1, PHOXB2, PGP9.5, TUBB3, PRPH, SLC18A3, NTRK2, TH*)	**FACS:** at vagal neural crest cells (p75+/HNK1+ double positive) **Immunofluorescence:** at hPSC stage (OCT4) at vagal neural crest spheroid stage (SOX10) at neuron stage β-3-tubulin, PRPH, SYP, PGP9.5, VAChT	Multielectrode Array/MEA live cell calcium imaging
	**Postganglionic parasympathetic neurons** [171, 172] hESCs and hiPSCs are differentiated into progenitor neural crest, purified and expanded through a 3D spheroid stage, and terminally differentiated to mature parasympathetic neurons. Considerations: ~20% contaminating cell types (other peripheral neurons, Schwann cells, endothelial cells, etc.)	**RT-qPCR:** at hPSC stage (*NANOG, OCT4*) at neural crest stage (*SOX10, AP2A*) at spheroid stage (*SOX10, S100B*) at neuron stage (*ASCL1, PHOX2B, PRPH, CHRNA3, CHAT, SLC18A, NPY2R*)	**Immunofluorescence:** at neural crest (SOX10, AP2a) at neuron stage (ChAT, VAChT, CHRNB4, NPY2R, PRPH, TUJ1)	Multielectrode Array/MEA
	**Parasympathetic-like neurons** [173] hESCs and hiPSCs are formed into embryoid bodies, further cultured to autonomic neural crest progenitors, and terminally differentiated into parasympathetic-like neurons. Considerations: conflicting results suggesting parasympathetic-like neurons are not entirely parasympathetic in character, no detection of acetylcholine release in culture media, and the embryoid body method of induction is inefficient because it yields highly heterogeneous cell populations	**scRNA-seq:** at neural crest stage (*PAX3, B3GAT1, NGFR, SOX10*) at neuron stage (*ASCL1, PHOX2A, PHOX2B* autonomic nervous system markers)	**Immunofluorescence:** at neural rest progenitor stage (NGFR and PHOX2B) at vagal neural crest spheroid stage (SOX10) at neuron stage (β-3-tubulin, PRPH, SYP, PGP9.5, VAChT) at parasympathetic neuron-like stage (ChAT and PHOX2B)	Multielectrode Array/MEA live cell calcium imaging
Sympathetic	**Sympathetic neurons** [174] hESCs and hiPSCs are differentiated into neuromesodermal progenitor cells, which then give rise to trunk neural crest cells, and finally terminally differentiated to sympathetic neurons at high purity (~80%). Considerations: characterisation of the sympathetic neurons and contaminating cell types is limited, a sympathetic neuron protocol is developed in floating spheroid cultures, and limited functionality assessments	**RT-qPCR:** at neural crest stage (*PHOX2B, SOX1, PAX6, SOX10, FOXD3, HOXB1, HOXB2, HOXB4, HOXB6, HOXB8, HOXC9*)	**Immunofluorescence:** at trunk neural crest stage (SOX10, PHOX2B) at sympathetic neuron stage (PRPH, TH, DBH, PHOX2B)	measured release of noradrenaline with and without KCl treatment
	**Post-ganglionic sympathetic neurons** [175, 176] hESCs and hiPSCs are differentiated into neural crest cells, purified to sympathoblast stage in a spheroid culture, and terminally differentiated into sympathetic neurons at high purity (~80%). Considerations: low numbers of contaminating smooth muscle cells, fibroblasts, and placode-derived non-neural cells.	**RT-qPCR:** at neural crest stage (*SOX10, TFAP2A*) at sympathoblast stage (*ASCL1, PHOX2B, PRPH, GATA3, HAND2, HOXB2, HOXB5, HOXB7, HOXC9, HOXA10*) at sympathetic neuron stage (*Ascl1, PHOX2B, PRPH, CHRNA3, CHRNB4, VMAT1, ADRA2A, ADRB2, NET, TH, AAAD, DBH, MAO-A*)	**ELISA:** (noradrenaline) **FACS:** sorting at neural crest stage (CD49d+ and SOX10+) **Immunofluorescence:** at neural crest stage (SOX10) at neuron stage (PRPH, CHRNA3, CHRNB4, TUJ1, TrkA, a2ADR, b2ADR, NET, VMAT1, VMAT2, TH, cFos, NPY)	Multielectrode Array/MEA live cell calcium imaging
	**Sympathetic neurons** [177] hESCs and hiPSCs are differentiated to derive sympathoadrenal progenitors through neural epithelial cells and trunk neural crest stem cells, and finally terminally differentiated into sympathetic neurons at high efficiency (>90%). Considerations: the sympathetic neuron protocol is not applied towards disease modelling, and contaminating cell types are not investigated	**RT-qPCR:** at neuroepithelial cell stage (*NANOG, KLF4, POU5F1, SOX1, SOX2, PAX6*) at trunk neural crest stage (*NGFR, SOX10, AP2a, PAX3, HOXB5, HOXB7, HOXC5, HOXD9*) at sympathoadrenal progenitor stage (*HAND2, ASCL1, GATA3, PHOX2B*) Bulk RNA-seq-based transcriptomic profiling of sympathetic neurons	**ELISA:** (noradrenaline and dopamine) **FACS:** sorting at trunk neural crest stage (p75NTR+ and HNK1+) sympathoadrenal progenitor stage (GD2+, p75NTR+) **Immunofluorescence:** at neuroepithelial cell stage (SOX1, SOX2, PAX6, Nestin, and Brachyury T) at trunk neural crest stage (SOX10, HOXC9, HOXC8) at sympathoadrenal stage (ASCL1, HAND2, PHOX2B, GATA3) at neuron stage (PRPH, TH, noradrenaline, PHOX2B, CgA, DBH, TUJ1)	via live cell calcium imaging whole-cell patch-clamp analysis
	**Sympathetic-like neurons** [173] hESCs and hiPSCs are formed into embryoid bodies, further cultured to autonomic neural crest progenitors, and terminally differentiated into sympathetic-like neurons. Considerations: no detection of noradrenaline release in culture media, and the embryoid body method of induction is inefficient because it yields highly heterogeneous cell populations	**scRNA-seq:** at neural crest stage) (*PAX3, B3GAT1, NGFR, SOX10*) at neural stage (*ASCL1, PHOX2A, PHOX2B* autonomic nervous system markers; *GATA2, GATA3, TH, PHOX2B, DDC* sympathetic neuron markers)	**Immunofluorescence:** at neural rest progenitor stage (NGFR and PHOX2B) at vagal neural crest spheroid stage (SOX10) at neuron stage (β-3-tubulin, PRPH, SYP, PGP9.5, VAChT) at neuron stage (TUJ1, PRPH, TH, and DBH)	Multielectrode Array/MEA live cell calcium imaging

## Conclusions

Looking forward, advances in modelling pulmonary nerve pathophysiology will benefit from developments in both xenogeneic and human in vitro approaches. Emerging platforms that enable functional interactions between neuronal terminals and target cells, such as 3D Matrigel-based innervation systems [183], compartmentalised co-culture models separating neuronal cell bodies from effector cells (e.g., fibroblasts) [184], and multicompartmental microfluidic devices [185] provide advanced platforms to investigate clinically relevant cell types and their interactions with neurons. In parallel, emerging evidence for organ-specific neuronal phenotypes [186] may inform the development of more physiologically relevant, lung-specific hPSC-derived neuronal cell populations. The development of robust in vitro models is essential to maximise the value of precious lung disease-relevant human samples, such as bronchoalveolar lavage fluid [187], and to enhance the precision and translational relevance of downstream experimental readouts.

Clinical insights may also be gained from perturbed pulmonary innervation, including targeted lung denervation [188, 189], thoracoscopic lung resection [190], vagus nerve stimulation [191], and lung transplantation, which results in near-complete denervation and may provide a framework for examining its relationship to fibro-inflammatory complications, as seen in chronic lung allograft dysfunction [192].

Together, these experimental and clinical platforms could support therapeutic strategies targeting neuronal signalling [58], neuro -immune pathways [48], and tumour-associated neural circuits [56, 193], collectively driving this interdisciplinary field of pulmonary-neuro pathophysiology towards clinical translation.

## Key References

- Almanzar N, Yang D, Xia J, Udit S, Joshi P, Adhikari S, et al. Vagal TRPV1(+) sensory neurons protect against influenza virus infection by regulating lung myeloid cell dynamics. Sci Immunol. 2025;10(110):eads6243. doi: 10.1126/sciimmunol.ads6243.
  - ○ In a murine influenza A model, chemical and genetic ablation of TRPV1 + sensory neurons enhances protective myeloid interferon signalling and reduces pathogenic neutrophil accumulation. Highlights context-dependent roles of sensory neurons during acute infection (protective role).
- Baral P, Umans BD, Li L, Wallrapp A, Bist M, Kirschbaum T, et al. Nociceptor sensory neurons suppress neutrophil and gammadelta T cell responses in bacterial lung infections and lethal pneumonia. Nat Med. 2018;24(4):417–26. doi: 10.1038/nm.4501.
  - ○ In a murine model of acute Staphylococcus aureus pneumonia, TRPV1 + sensory neurons suppress protective innate immunity via CGRP release, limiting neutrophil recruitment and cytokine responses. Highlights context-dependent roles of sensory neurons during acute infection (pathogenic role).
- Ishikawa G, Peng X, Ghincea A, McGovern J, Zielonka J, Jeevanandam A, et al. A Nerve-Fibroblast Axis in Mammalian Lung Fibrosis. bioRxiv. 2025. doi: 10.1101/2024.09.09.611003.
  - ○ A recent study, defining a pathogenic adrenergic nerve-fibroblast axis in pulmonary fibrosis, using 3D mapping and genetic manipulation of sympathetic innervation in bleomycin-injured mice, human fibrosis samples, nerve-fibroblast co-culture and terazosin-treated human PCLS to show that sympathetic signalling promotes fibroblast differentiation and fibrotic remodelling.

## Data Availability

No datasets were generated or analysed during the current study.
